# Routine preoperative assessment for cataract surgery is a source of frustration for primary care providers

**DOI:** 10.1186/s12913-024-11484-0

**Published:** 2024-09-17

**Authors:** Donna Ron, Michael Zegans, Catherine L. Chen

**Affiliations:** 1 Department of Community and Family Medicine, Dartmouth Health and Geisel School of Medicine at Dartmouth, Lebanon, NH USA; 2 Section of Ophthalmology, Dartmouth Health and Geisel School of Medicine at Dartmouth, Lebanon, NH USA; 3 University of California San Francisco Francis I. Proctor Foundation, San Francisco, CA USA; 4 Department of Anesthesia & Perioperative Care, University of California San Francisco, San Francisco, CA USA; 5 Philip R. Lee Institute for Health Policy Studies at UCSF, San Francisco, CA USA; 6 Department of Anesthesiology and Perioperative Medicine, Dartmouth Health, Lebanon, NH USA

**Keywords:** Cataract extraction, Cataract surgery, Low-value care, Physicians, Primary Care, Medicare, Preoperative clearance, Preoperative History & Physical, Preoperative Assessment, Routine preoperative testing, Qualitative research

## Abstract

**Background:**

Cataract surgery is one of the most common surgical procedures performed in older adults in the United States and is generally considered to be extremely low-risk. As of 2019, routine preoperative evaluation within 30 days of surgery is no longer mandated by the United States of America (USA) Centers for Medicare & Medicaid Services (CMS) for ambulatory surgery centers, but it is unclear how primary care providers perceive this change.

**Methods:**

We performed a qualitative analysis of semi-structured interviews with six primary care providers to explore primary care providers’ perspectives on routine preoperative assessment for cataract surgery.

**Results:**

Primary care providers commented on the large number of referrals they receive for preoperative assessment before cataract procedures. The analysis revealed an overarching sentiment of resentment over the time, effort, and resources expended on these assessments. Themes included the lack of awareness of the updated regulations that no longer require a history and physical to be completed within 30 days and the perception of a universal lack of medical necessity to perform preoperative assessment for cataract surgery. Providers also commented on the strain on limited resources and the burden on patients. The relationship between specialties and professional roles emerged as another important theme.

**Conclusions:**

Referrals for preoperative clearance for cataract surgery continue to burden providers, patients, and the health system, and represent an opportunity to streamline care in this patient population.

**Supplementary Information:**

The online version contains supplementary material available at 10.1186/s12913-024-11484-0.

## Background

Cataract is one of the leading causes of blindness worldwide but is easily amenable to surgical treatment [1, 2]. Cataract surgery is one of the most common surgical procedures performed in older adults in the United States, and is generally considered to be extremely low-risk [3–5]. Studies have shown that routine preoperative medical testing (such as blood work and electrocardiograms) is inconvenient for patients, increases healthcare costs, and can harm patients by unnecessarily delaying surgery without improving surgical outcomes [6, 7]. Since this defines low-value care, professional society guidelines have recommended avoiding routine preoperative medical testing in this patient population [8–10]. More recently, experts have suggested that routine preoperative history and physical (H&P) evaluation before cataract surgery could also be reduced without negative consequences to patients [11, 12].

Historically, regulatory guidelines from The United States Centers for Medicare and Medicaid Services (CMS) mandated that an H&P be completed within 30 days before surgery performed in an ambulatory surgery center (ASC) [13]. In late 2019, CMS removed this requirement, deferring “to the ASC policy and operating physician’s clinical judgment to ensure that patients receive the appropriate pre-surgical assessments tailored to the patient and the type of surgery being performed.” [14] However, it is unclear whether this ruling has impacted the utilization of routine preoperative evaluation of cataract surgery patients.

To illustrate how this CMS ruling may have influenced practice patterns associated with routine preoperative evaluation before cataract surgery from the perspective of the primary care providers (PCPs), we report data from six semi-structured interviews.

## Methods

The interview excerpts we present here were collected as part of a mixed methods study on preoperative communication between anesthesia, surgery and PCPs in northern New England [15, 16]. The study protocol was reviewed by the Dartmouth Hitchcock Medical Center (DHMC) Institutional Review Board (IRB) and determined to be exempt. Before enrolling in the study, participants reviewed an information sheet which included the elements of informed consent as dictated by the Dartmouth-Hitchcock IRB and consent was implied by participation in the survey and/or interview. The parent study included anesthesia providers, surgeons, PCPs and older surgical patients; however, the findings discussed in the current manuscript were obtained exclusively from PCPs. The study is reported according to the COnsolidated criteria for REporting Qualitative research (COREQ) reporting guidelines [17].

The first portion of the parent study consisted of an online survey distributed to all eligible clinicians and a random sample of older surgical patients. PCPs with 2 or more older patients seen at the DHMC Perioperative Care Clinic in 2022 were recruited by email or telephone from January through May 2023. These included PCPs from larger academic centers such as DHMC and the Veterans Affairs Medical Center as well as critical access hospitals in northern New Hampshire and small rural primary care practices across the region. Clinician surveys included a question regarding their willingness to be interviewed. Using consecutive sampling, we reached out via email to all clinicians who expressed willingness and interviewed those that responded.

The surveys and interview guides were developed through observation of the workflow of the perioperative clinic, meetings with perioperative physicians and nurses, and input from representatives from diverse stakeholder groups. Guidance from qualitative and mixed-methods experts, cognitive interviews and pilot tests informed further revisions as indicated. More detailed information on the survey and interview guide development process is included in a separate manuscript describing the main study results (currently in press). The semi-structured interview guides were written with the aim of exploring clinician experiences and perspectives on communication and role distribution between different disciplines in the preoperative setting. They provided sample questions for each domain, along with optional follow-up questions designed to elicit further details and examples. When clinicians deviated from the sample questions, follow-up questions were not restricted to those listed in the guide. The original interview guides (available in the Supplementary file) did not include any questions regarding preoperative assessment for cataract surgeries – several PCPs brought up this issue unprompted and the relevant interview excerpts were compiled for separate analysis.

The first author (DR), who is an anesthesiologist and researcher with training and experience in qualitative research methods, conducted all interviews via videoconferencing, telephone, or in person. Interviews took place from January through May 2023 and lasted between 32 and 86 min (47 min per interview on average). At the beginning of each interview, participants were made aware of the interviewer’s background and the research objectives, either through a brief explanation or by reviewing the information sheet provided with the interview invitation. After each session, the interviewer took field notes to document impressions of the overall atmosphere, any technical issues or interruptions, and the main insights gathered during the interview. WebEx videoconferencing software was used to generate interview recordings and automated transcripts. The interviewer subsequently reviewed the automated transcripts for accuracy, referencing the recordings to correct any mistakes and eliminate identifying information. To honor the participants’ time, transcripts were not sent back to clinicians for revisions.

The de-identified interview transcripts were analyzed by DR and a research assistant using a combination of the Sort and Sift, Think and Shift method [18] aided by Microsoft Office software, and thematic analysis aided by ATLAS.ti software. The theoretical framework for the qualitative analysis incorporated core principals from the phenomenology and case study qualitative traditions [18]. Analysis tools included memoing, identifying powerful quotations, creating episode profiles, diagramming, and iterative inductive coding of all transcripts. PCP commentary on preoperative assessments for cataract surgery was coded as such and subsequently extracted by DR for separate analysis. The relevant excerpts were then analyzed by the first and last authors (DR and CLC), and consensus was reached regarding codes and themes through discussion among the research team (DR, CLC, MZ).

## Results

Twenty clinicians participated in interviews for the parent study: four anesthesia providers, seven surgeons, and nine PCPs. The PCPs ranged in age between 30 and 60 + and included both male and female participants. PCP specialties included internal medicine, family medicine, and geriatrics. Of the clinicians who participated in the parent study, six PCPs (one advanced practice provider and five physicians) brought up the frequent preoperative assessments they perform for cataract procedures (per PCP6, *“[cataract] is the thing we see the most in terms of preoperative assessments”*). Despite not being specifically asked about preoperative assessments for cataract surgery, PCPs seemed eager to voice their frustrations on the topic. It came up repeatedly over multiple domains of the interview and was often accompanied by stronger emotional undertones as indicated by changes in tone of voice and hand gestures. The unsolicited, repeated mentions and emotionally charged discourse suggested that PCPs feel very strongly about this topic, and that these PCP utterances merit a separate standalone analysis.

Analysis of the interview excerpts revealed five main themes, discussed in further detail below. Themes included a lack of awareness of the updated regulations, a lack of medical necessity to perform preoperative assessments for cataract procedures, the burden these assessments impose on patients, the strain on limited primary care resources, and PCP perspectives on interprofessional relationships and responsibilities. Table 1 summarizes the main themes and highlights representative excerpts.

**Table 1:** Main themes and representative excerpts^a^

Themes	Quotations
Lack of awareness of regulations	**PCP5**: **“Apparently CMS requires a Pre-op for cataract surgery**,** which is insane as far as most of us are concerned**…but they all are supposed to go to pre-op for some unknown -we believe it’s somewhere out in CMS land - reason, and **it’s a waste of our time**… And the such and such [city] **eye clinic wants an EKG and blood work** for their cataracts, **which drives us crazy** too. Seriously. For what reason? I don’t know.” **PCP7**: “So a Pre op evaluation needs to be done 30 days prior to the surgery. It’s not the preoperative evaluation that needs to be done - **an H&P needs to be on the chart within 30 days of the surgery.**”
Lack of medical necessity	**PCP2**: “Interestingly, the one that **we get consistent referrals for**, and that’s what my experience has been here, too, was **most of my pre-ops actually dealt with cataracts**,** which technically you don’t really even need…**It’s a very simple surgery, and yet I will have to do pre-op, and then some–particularly outside ophthalmologists–would want us to do an EKG.” **PCP5**: “**I use it for anything else**. Like, let’s talk about your blood pressure, and did you have a pneumococcal vaccine, and all the other things. **Because I’m going to rubber stamp everybody for cataract surgery**. I mean, it could be the worst heart patient and if you really want cataract surgery, I’m going to say, fine…There’s zero circumstances that we would keep somebody out of cataract surgery.” **PCP7**: “I do a number of pre-op evaluations for cataract surgery. **Um**,** they really aren’t necessary.** Cataract is a minor surgery, minor elective surgery. I don’t feel I add much. Sometimes they even ask for an EKG. I don’t know whether that adds anything.”
Burden on patients	**PCP4**: “Yesterday, we saw somebody with a March 29th cataract. And then her next one is May 11th, and **she’s gonna have to come back in the middle of April for the second** [pre-op]. Totally healthy - We did zero things.” **PCP2**: “…then the craziest part was…so, they did one eye - like, left eye, I did a pre op for the left eye because the date was like, 6 weeks or something. You know, there was a lag of more than 4 weeks from the last time I saw the patient, they wanted me to do another pre-op for the right eye. [laughing] …**It was inconvenient for a lot of my patients. I mean**,** they travel 2 hours**. Finally, I got hold of one of our more senior cataract surgeons here and I asked him, hey, can you help me understand why? I’m doing another pre-op in the span of 6 weeks? And they said that there was this rule that if you, at least from their end, that they need to have the patients be seen if it’s more than 4 weeks. So, from then on, though, it had gotten better in the sense that right **now they’re really timing the surgery that it’s at least 3 weeks apart. So that you only do one pre-op**.”
Strain on limited resources	**PCP8**: “…a lot of them get unnecessary pre-ops, and **I think it clogs up our system a lot.** It’s a lot of real estate on people’s schedules that doesn’t necessarily need to be taken up.” **PCP4**: “**It is a big waste of our time**…We pre-op every cataract and **it’s a huge time suck for everybody** …[we] don’t need to know every cataract surgery that’s happening.”
Relationship between specialties and professional roles and responsibilities	**PCP7**: “…This allows the ophthalmologist to not do an H&P. **So**,** essentially**,** we’re being used as their intern.** That’s what’s going on. There’s not a medical reason for it. Now, I get paid, but it’s not useful. **It takes that duty off the surgeon**. Now, the surgeon would claim they don’t get paid to do an H&P. But they do, because surgery, you’re paid a global fee for the surgery, which includes the H&P^b^ and any post-op. So, what they’re doing is **they’re offloading a piece of their global fee to another person**.”

Representative quotes from the six PCPs in this study, derived from a larger mixed-methods study that included nine PCPs. The PCP IDs reflect their anonymized identification number from the parent study [15].

Preoperative care by the surgeon, such as a preoperative H&P, is included in the global surgery fee if performed on the day of surgery or up to one day prior to surgery. (source: https://www.cms.gov/files/document/mln907166-global-surgery-booklet.pdf)

Abbreviations: CMS = Centers for Medicare & Medicaid Services, EKG = Electrocardiogram, H&P = History and physical, PCP = Primary care provider.

### Lack of awareness of regulations

PCPs described the preoperative H&P as a “rule” imposed on them by clinics, surgery centers, or regulatory requirements. None of the PCPs interviewed reported awareness of the updated regulations regarding preoperative assessment, and many cited the former CMS requirement for a preoperative H&P to be obtained within 30 days of surgery at an ASC. Others noted that an H&P was required but were unsure what the source of the requirement was. PCPs also mentioned eye clinics and ophthalmologists requiring electrocardiograms or labs prior to cataract surgery, “*which drives us crazy too. Seriously. For what reason? I don’t know*.” (PCP5).

### Lack of medical necessity

A predominant theme that emerged from PCP commentary was the perceived lack of medical necessity to perform preoperative assessment for cataract surgery. PCPs asserted that cataract surgery presents a low perioperative risk, and therefore should not routinely require referral for preoperative evaluation. Most PCPs seemed to feel that these assessments and any testing ordered represent low-value care. As PCP5 commented, “*Because I’m going to rubber stamp everybody for cataract surgery*.”

### Burden on patients

Another theme PCPs spoke of was the burden imposed on patients resulting from redundant preoperative assessments. Coming to the PCP office for a preoperative assessment for cataract surgery was especially burdensome when two cataract procedures occurring more than 30 days apart resulted in the patient returning for a second appointment just a few weeks after the first. PCP2 reported that in an attempt to reduce the burden on PCPs and patients, “*right now they’re really timing the surgery so that it’s at least 3 weeks apart. So that you only do one pre-op*”. Some PCPs described attempts to make the most of this situation by incorporating screening or other tasks into the preoperative appointment.

### Strain on limited resources

Perioperative challenges related to limited primary care resources were a recurring theme in our qualitative interviews across disciplines. Clinicians described a severe shortage of PCPs, with many patients unable to establish care or book primary care appointments. PCPs reported that the most frequent preoperative evaluations they perform are for cataract procedures, and that these took up a lot of time on their already overflowing schedules. Although PCPs expressed wanting to know about high-risk procedures taking place on their patients, they felt it was less essential for them to be aware of upcoming cataract surgeries. Per PCP8, “*it’s a lot of real estate on people’s schedules that doesn’t necessarily need to be taken up.”*

### Relationship between specialties and professional roles and responsibilities

Some of the interview questions related to the inter-specialty distribution of perioperative roles and responsibilities among anesthesia, surgery, and primary care. PCPs had differing opinions regarding the role of the surgeon, with some describing situations in which they felt surgeons were “dumping” less preferred or less profitable tasks on PCPs. An overarching sentiment of resentment was present and seemed to mainly target the CMS or hospital regulations, which PCPs felt were requiring them to waste time on low-value care, but some of it also targeted surgeons. PCP7 questioned why ophthalmologists were delegating the preoperative H&P to PCPs. The sentiment expressed seemed to be a feeling of being taken advantage of, as PCP7 concluded: “*So*,* essentially*,* we’re being used as their intern*.”

## Discussion

We performed a qualitative analysis of excerpts from interviews with six primary care providers to explore their perspectives on routine preoperative assessment for cataract surgery. We found that despite CMS regulations no longer requiring a history and physical to be completed within 30 days in patients having outpatient surgery at an ASC, PCPs expressed their ongoing dissatisfaction with the frequent referrals of cataract surgery patients for preoperative clearance. Themes included the lack of awareness of the updated regulations and the perception of a universal lack of medical necessity to perform preoperative assessment for cataract surgery. Providers also commented on the strain on limited primary care resources and the burden on patients. The relationship between specialties and professional roles emerged as another important theme, with PCPs expressing frustration over the perception that surgeons are shifting responsibilities onto them. Overall, PCPs expressed an overarching sentiment of resentment over the time, effort, and resources expended on these assessments.

There are several reasons why the practice of referring patients to primary care for preoperative clearance prior to cataract surgery may be lagging behind the change in CMS regulations. Our results suggest that there is a lack of awareness of this change in regulations among clinicians and institutions. Even among those who are aware, there may be ongoing resistance to changing longstanding practice patterns [19]. For example, some physicians believe that cataract surgery is an important opportunity to refer patients who may not previously have had regular contact with their PCP into medical care [20]. In addition, there are certain circumstances that might merit additional input from the patient’s PCP. When topical analgesia is not adequate for cataract procedures that are expected to be prolonged or complicated (e.g., dense nucleus, small pupil, zonule compromise, or exfoliation syndrome) and more invasive analgesic techniques (e.g., retrobulbar or peribulbar blocks) are required, input regarding anticoagulation cessation risk helps inform treatment decisions. A preoperative assessment might also be useful for patients at risk for intraoperative conversion to general anesthesia, such as those with airway compromise, anxiety disorders or PTSD. For elderly patients with frailty, the preoperative assessment could also help the patient and surgeon decide whether to delay surgery pending optimization of chronic conditions. Finally, ophthalmologists who practice in rural settings may feel more comfortable operating on high-risk patients after consulting with the PCP, since freestanding ASCs may not have proximity to acute care resources. However, according to the PCPs we interviewed, many patients are indiscriminately referred for preoperative assessment, even when they have recently had cataract surgery on the other eye or have recently seen their PCP for an annual physical. Therefore, by decreasing the frequency of requests for patients who have been examined recently or are routinely followed by their PCP, we have an opportunity to reduce the burden on patients and physicians.

A large case series from ophthalmologists performing office-based cataract surgery suggests that cataract surgery can safely be performed in selected patients without a preoperative assessment by either the PCP prior to surgery or by anesthesia-trained clinicians on the day of surgery, with the caveat that most patients in this cohort were categorized as low-risk (ASA 1 and 2 according to the American Society of Anesthesiologists physical status classification guidelines) [21] and may not be representative of patients requiring more preoperative care coordination [22]. Others have shown that there is wide geographic variation in the use of routine preoperative assessment in Medicare beneficiaries undergoing cataract surgery [20]. In addition, the Society for Ambulatory Anesthesia’s recently issued guidelines for cataract surgery recommend that, with very few exceptions, most patients can safely proceed without further workup [4]. Therefore, for stable patients, a more streamlined approach could allow PCPs the option to communicate directly to the ophthalmologist regarding the patient’s fitness for surgery without performing a full H&P. This alternative approach would reduce the current burden of routine preoperative assessment in this patient population while ensuring that higher-risk patients or patients who are not already engaged with the healthcare system would still have the opportunity to be seen by their PCP to manage conditions that are outside the scope of the ophthalmologists’ expertise. Additional studies that evaluate the association between routine preoperative assessment, cataract surgery outcomes, and the risk of perioperative adverse events, regardless of the sedation approach used, would help strengthen the evidence base [12].

While our work establishes the burden felt by PCPs in the requirements for preoperative physicals, gathering and analyzing input from ophthalmologists, anesthesiologists and other stakeholders, which our team is pursuing, will be necessary in crafting solutions to the valid concerns of PCPs. Ophthalmologists are often many years away from their general medical training and may feel uncomfortable determining a patient’s medical stability or appropriately testing and organizing follow-up examinations if medical problems are identified. Concern also exists that a patient might misinterpret a brief medical screening by an ophthalmologist as equivalent to an evaluation by a generalist. Time management and resource utilization are also concerns if the medical evaluations are being done on the day of surgery (be it by ophthalmologists or anesthesiologists) with regards to workflow implications as well as a higher rate of case cancellations from patients not being optimized for surgery [23]. Since ACSs are often judged on the number of medical transfers from their facilities, ASC directors may be reluctant to have potentially unstable patients undergoing surgery at the ASC [24].

Limitations of this study include the relatively small sample size originating from one geographic area and the lack of input from other important stakeholders on the topic of preoperative assessment. In addition, since the data originated from a parent study not focused on this specific topic, our interview guides did not contain any specific questions on the topic, which would have enabled more detail or nuance to be obtained. Since our quotes originated from PCPs who brought up the subject unprompted, they likely reflect the perspectives of those who felt most strongly about the burden imposed by these referrals. Had we included specific questions regarding preoperative assessments for cataract surgery, we may have been able to obtain more information regarding situations in which PCPs feel that a preoperative referral is merited, such as among patients undergoing more complicated or invasive cataract procedures.

Despite these limitations, our study suggests that the updated CMS regulations offer an opportunity to reduce the burden of low-value care in the United States, and in turn, improve access to care for those who need it. Tailoring requests for preoperative assessment rather than continuing to routinely refer all cataract surgery patients for preoperative assessment will provide ophthalmologists with the necessary support in perioperative management of patients’ chronic medical conditions while ensuring that limited resources are utilized efficiently. This is a critical task since the majority of patients undergoing cataract surgery in the United States are over 65 years of age. With nearly 99% of eligible US adults over age 65 currently enrolled in Medicare [25] and every member of the Baby Boom generation soon to reach Medicare eligibility, [26] the number of older adults needing cataract surgery will continue to increase, which will categorically increase the time and resources devoted to these extra office visits and their associated routine lab tests and cardiac work-up [6, 27, 28]. At the same time, primary care providers are an increasingly scarce resource in the United States and much has been written regarding the frustrations of primary care physicians leading to burnout and a mass exodus of clinicians from clinical medicine, especially after the challenges faced during the COVID-19 pandemic [29–31]. Therefore, every effort must be made to preserve their ability to deliver essential primary care to an aging population.

In summary, based on qualitative interviews with PCPs, routine referrals for preoperative clearance in cataract surgery patients continue to burden PCPs, patients, and the health system. By reducing the volume of requests for routine preoperative clearance and identifying innovative solutions to ensure that high-risk patients are appropriately optimized before undergoing cataract surgery, we have an opportunity to improve access to vital primary care resources and decrease unnecessary demands on primary care physicians.

## Electronic supplementary material

Below is the link to the electronic supplementary material.

Supplementary Material 1

Supplementary Material 2

## Data Availability

The datasets used and/or analysed during the current study are available from the corresponding author on reasonable request.

## References

[1] Fang R, Yu Y-F, Li E-J, et al. Global, regional, national burden and gender disparity of cataract: findings from the global burden of disease study 2019. BMC Public Health. 2022;22(1):2068. 10.1186/s12889-022-14491-0. 36369026 10.1186/s12889-022-14491-0 PMC9652134

[2] Liu YC, Wilkins M, Kim T, Malyugin B, Mehta JS, Cataracts. Lancet (London England). 2017;390(10094):600–12. DOI: S0140-6736(17)30544-5 [pii]. 28242111 10.1016/S0140-6736(17)30544-5

[3] Schein OD, Cassard SD, Tielsch JM, Gower EW. Cataract surgery among Medicare beneficiaries. Ophthalmic Epidemiol. 2012;19(5):257–64. 10.3109/09286586.2012.698692. [doi]. 22978526 10.3109/09286586.2012.698692 PMC4313376

[4] Sweitzer B, Rajan N, Schell D, Gayer S, Eckert S, Joshi GP. Preoperative care for cataract surgery: the Society for ambulatory anesthesia position Statement. Anesth Analg. 2021;133(6):1431–6. 10.1213/ane.0000000000005652. (In eng). 34784329 10.1213/ANE.0000000000005652

[5] National Eye Institute. Eye Health Data and Statistics: Cataract Tables. (https://www.nei.nih.gov/learn-about-eye-health/eye-health-data-and-statistics/cataract-data-and-statistics/cataract-tables)

[6] Chen CL, Clay TH, McLeod S, Chang HP, Gelb AW, Dudley RA. A revised Estimate of costs Associated with Routine Preoperative Testing in Medicare Cataract patients with a Procedure-Specific Indicator. JAMA Ophthalmol. 2018;136(3):231–8. 10.1001/jamaophthalmol.2017.6372. (In eng). 29346472 10.1001/jamaophthalmol.2017.6372 PMC5885904

[7] Chen CL, McLeod SD, Lietman TM et al. Preoperative Medical Testing and Falls in Medicare Beneficiaries Awaiting Cataract Surgery. Ophthalmology 2020 (In eng). 10.1016/j.ophtha.2020.09.013 10.1016/j.ophtha.2020.09.013 PMC8443237 32926912

[8] Committee on S, Practice P, Apfelbaum JL, et al. Practice advisory for preanesthesia evaluation: an updated report by the American Society of Anesthesiologists Task Force on Preanesthesia evaluation. Anesthesiology. 2012;116(3):522–38. 10.1097/ALN.0b013e31823c1067. [doi]. 22273990 10.1097/ALN.0b013e31823c1067

[9] American Academy of Ophthalmology. Routine pre-operative laboratory testing for patients scheduled for cataract surgery, Clinical Statement. (http://www.aao.org/clinical-statement/routine-preoperative-laboratory-testing-patients-s)

[10] Fleisher LA, Fleischmann KE, Auerbach AD et al. 2014 ACC/AHA guideline on perioperative cardiovascular evaluation and management of patients undergoing noncardiac surgery: executive summary: a report of the American College of Cardiology/American Heart Association Task Force on practice guidelines. Developed in collaboration with the American College of Surgeons, American Society of Anesthesiologists, American Society of Echocardiography, American Society of Nuclear Cardiology, Heart Rhythm Society, Society for Cardiovascular Angiography and Interventions, Society of Cardiovascular Anesthesiologists, and Society of Vascular Medicine Endorsed by the Society of Hospital Medicine. Journal of nuclear cardiology: official publication of the American Society of Nuclear Cardiology 2015;22(1):162–215. 10.1007/s12350-014-0025-z [doi]. 10.1007/s12350-014-0025-z 25523415

[11] Fleisher LA. Preoperative consultation before cataract surgery: are we choosing wisely or is this simply low-value care? JAMA Intern Med. 2014;174(3):389–90. 10.1001/jamainternmed.2013.12298. [doi]. 24366215 10.1001/jamainternmed.2013.12298

[12] Merali FI, Schein OD. Preoperative evaluations for cataract surgery are routine but anachronistic. JAMA Ophthalmol. 2018;136(3):239–239. 10.1001/jamaophthalmol.2017.6278. 29346487 10.1001/jamaophthalmol.2017.6278

[13] Centers for Medicare & Medicaid Services. Clarifications to, Appendix L. Ambulatory Surgical Center Interpretive Guidelines – Comprehensive Medical History and Physical (H&P) Assessment and Anesthetic Risk and Evaluation. Department of Health & Human Services (DHHS). May 13, 2011 (https://www.hhs.gov/guidance/sites/default/files/hhs-guidance-documents/R71SOMA.pdf).

[14] Center for Medicare and Medicaid Services. Medicare and Medicaid Programs; Regulatory Provisions To Promote Program Efficiency, Transparency, and Burden Reduction; Fire Safety Requirements for Certain Dialysis Facilities; Hospital and Critical Access Hospital (CAH) Changes to promote Innovation, Flexibility, and improvement in Patient Care September 30, 2019 (https://www.govinfo.gov/content/pkg/FR-2019-09-30/pdf/2019-20736.pdf).

[15] Ron D, Gunn CM, Havidich JE, Ballacchino MM, Burdick TE, Deiner SG. Preoperative Communication between Anesthesia, Surgery, and Primary Care Providers for Older Surgical Patients. The Joint Commission Journal on Quality and Patient Safety 2024. 10.1016/j.jcjq.2024.01.006 10.1016/j.jcjq.2024.01.006 38360446

[16] Ron D, Ballacchino MM, Briggs A, Deiner SG. Clinician perspectives on the perioperative roles and responsibilities of anesthesia, surgery, and primary care. Am J Surg. 2024;115948. 10.1016/j.amjsurg.2024.115948 39245593

[17] Tong A, Sainsbury P, Craig J. Consolidated criteria for reporting qualitative research (COREQ): a 32-item checklist for interviews and focus groups. Int J Qual Health Care. 2007;19(6):349–57. 10.1093/intqhc/mzm042. 17872937 10.1093/intqhc/mzm042

[18] Maietta R, Mihas P, Swartout K, Petruzzelli J, Hamilton AB, Sort, Sift. Qualitative Rep. 2021;26(6):2045–60. 10.46743/2160-3715/2021.5013. (In English)., Think and Shift: Let the Data Be Your Guide An Applied Approach to Working With, Learning From, and Privileging Qualitative Data.

[19] Cabana MD, Rand CS, Powe NR, et al. Why don’t physicians follow clinical practice guidelines? A framework for improvement. JAMA: J Am Med Association. 1999;282(15):1458–65. DOI: jrv90041 [pii]. 10.1001/jama.282.15.1458 10535437

[20] Thilen SR, Treggiari MM, Lange JM, Lowy E, Weaver EM, Wijeysundera DN. Preoperative consultations for medicare patients undergoing cataract surgery. JAMA Intern Med. 2014;174(3):380–8. 10.1001/jamainternmed.2013.13426. [doi]. 24366269 10.1001/jamainternmed.2013.13426 PMC4167873

[21] American Society of Anesthesiologists Committee on Economics. ​Statement on ASA Physical Status Classification System. December 13, 2020 (https://www.asahq.org/standards-and-practice-parameters/statement-on-asa-physical-status-classification-system)

[22] Kugler LJ, Kapeles MJ, Durrie DS. Safety of office-based lens surgery: U.S. multicenter study. J Cataract Refract Surg. 2023;49(9):907–11. 10.1097/j.jcrs.0000000000001231. (In eng). 37276271 10.1097/j.jcrs.0000000000001231

[23] Ferschl MB, Tung A, Sweitzer B, Huo D, Glick DB. Preoperative clinic visits reduce operating room cancellations and delays. Anesthesiology. 2005;103(4):855–9. 10.1097/00000542-200510000-00025. (In eng). 16192779 10.1097/00000542-200510000-00025

[24] (CMS) TCfMMS. Ambulatory Surgical Center Quality Reporting (ASCQR) Program Measures. 2024.

[25] Bureau UC. Dual Health Insurance Coverage Declining For Adults Age 65 and Over. 2024 (https://www.census.gov/library/stories/2024/04/older-adults-health-coverage.html)

[26] Vespa JML, Armstrong DM. Demographic turning points for the United States: Population projections for 2020 to 2060. Washington, DC: US Census Bureau; 2020. pp. 25–1144.

[27] Chen CL, Lin GA, Bardach NS, et al. Preoperative medical testing in Medicare patients undergoing cataract surgery. N Engl J Med. 2015;372(16):1530–8. 10.1056/NEJMsa1410846. [doi]. 25875258 10.1056/NEJMsa1410846 PMC5536179

[28] Ganguli I, Lupo C, Mainor AJ, et al. Prevalence and cost of Care cascades after low-value preoperative electrocardiogram for cataract surgery in fee-for-service Medicare beneficiaries. JAMA Intern Med. 2019;179(9):1211–9. 10.1001/jamainternmed.2019.1739. (In eng). 31158270 10.1001/jamainternmed.2019.1739 PMC6547245

[29] Abbasi J. Pushed to their limits, 1 in 5 Physicians intends to leave practice. JAMA. 2022;327(15):1435–7. 10.1001/jama.2022.5074. 35353134 10.1001/jama.2022.5074

[30] Lawson E. The Global Primary Care Crisis. Br J Gen Pract. 2023;73(726):3. 10.3399/bjgp23X731469. (In eng). 36543556 10.3399/bjgp23X731469 PMC9799366

[31] TA H. All hands on deck needed to confront physician shortage crisis. American Medical Association; 2024.

