# Supplementary material for: Routine preoperative assessment for cataract surgery is a source of frustration for primary care providers
Source: BMC Health Serv Res. 2024 Sep 17;24:1087. doi: 10.1186/s12913-024-11484-0 (PMC11409469; doi:10.1186/s12913-024-11484-0)
Supplement: Supplementary file 1 — Supplementary Material 1 [file 12913_2024_11484_MOESM1_ESM.pdf]

Anesthesia Interview Guide

Thank you for your time and willingness to be interviewed to provide your valuable experience and insights for this research study. Is it ok for me to begin recording now?

Category	Interview Question	Relevant Objective	Follow-up Questions
**General questions about the interviewee**	1. How long have you been practicing?<br>**2.** Do you or have you practiced in other places in the past?	If yes, for the subsequent questions explore how their experiences have differed in other practice settings/locations	Which other places/settings?
**Perioperative communication experiences with PCPs**	1. How often do you encounter the need to communicate with primary care providers?<br>2. How do you usually convey information to PCPs within and outside of the DH system?<br>3. How often are you able to "close the loop" when communicating with PCPs and how is this most frequently achieved?<br>4. What challenges do you encounter when trying to convey or obtain information from primary care providers?<br>5. Have you ever been contacted by a PCP before surgery? If yes, please elaborate.	**Perioperative interdisciplinary communication experiences**	(1)Can you think of any examples of times you needed to communicate with PCPs?<br>(4)Examples of times when it would have been helpful to communicate with a PCP but you were unable to connect?
**Perioperative communication experiences with surgeons**	6. How do you usually convey information to surgery providers in the preoperative clinic setting?<br>7. What challenges do you encounter when trying to convey or obtain information from surgery providers?	**Perioperative interdisciplinary communication experiences**	(6)Can you think of any examples of times you talked to or corresponded with surgery providers during preoperative assessment that were positive or negative experiences?<br>(7)Examples of times when it would have been helpful to communicate with a surgeon but you were unable to connect?
**Importance of communication**	8. Do you think it's important to communicate with the PCP?		(8)Do you think it could improve patient outcomes?

**Communication preferences**	9. What are your preferred channels of communication about patients with other healthcare providers? (eg. Fax, telephone, email, mail, EMR)<br>10. Which channels of communication do you prefer to avoid, if any?	**How do anesthesia providers prefer to communicate?**	(9)How do you prefer to send info to other providers?<br>How do you prefer to request info?<br>Which channel of communication do you prefer to use to discuss a complex patient?
	11. Which pieces of information do you perceive as most important to convey to PCPs?<br>12. Which pieces of information do you perceive as most important to receive from PCPs?	Which information is important to communicate about	(11)Which pieces of information do you perceive as most important to convey to surgeons?<br>(12)Which pieces of information do you perceive as most important to receive from surgeons?
	13. What do you think about primary care doctors participating in preoperative multidisciplinary meetings about complex or high-risk patients?	Should we invite PCPs to participate in multidisciplinary meetings?	
	14. What are the main barriers that impede communication with surgery providers and PCPs?	Communication barriers	
	15. What do you wish was in place to facilitate communication with surgery and primary care providers before surgery?	**Potential strategies to enhance communication**	Do you have any suggestions or things you think should be done?
**Preoperative assessment and optimization**	16. How much time do you typically spend on gathering information about patients during preoperative assessment?<br>17. What are the most frequent tests you order when performing preop assessments, and who are the specialists you consult most frequently?		(17)How does your communication with these consultants compare with your communication with PCPs and surgeons?
	18. How do you perceive the perioperative clinic's responsibilities in preoperative assessment, optimization and preparation?	Perspectives of preoperative assessment, distribution of preoperative responsibilities between anesthesia, primary care and surgery.	

	19. How do you perceive the PCP's responsibilities in preoperative assessment, optimization and preparation?<br>20. How do you perceive the surgeon's responsibilities in preoperative assessment, optimization and preparation?		
**Concluding/Final thoughts**	Are there any other thoughts you would like to share about perioperative communication between PCPs, anesthesia providers and surgeons?		

PCP Interview Guide

Thank you for your time and willingness to be interviewed to provide your valuable experience and insights for this research study. Is it ok for me to begin recording now?

Category	Interview Question	Relevant Objective	Follow-up Questions
**General questions about the interviewee**	1. Review of demographic info from the survey<br>2. How long have you been practicing?<br>**3.** Do you or have you practiced in other places in the past?	If yes, for subsequent questions explore how experiences differed in other practice settings/locations	Which other places/settings?
**Awareness of surgeries**	4. How often are you notified of impending surgical procedures for your patients?<br>5. When do you typically find out about them?<br>6. How do you usually find out about them?<br>Were there times where you wished you had known earlier?	How often do PCPs know about surgeries and how and when do they find out about them. Do they want to know earlier? Should we be notifying them?	Would you prefer to know more frequently/earlier or in certain situations?<br>When and how is it helpful to know about surgeries?<br>How would you want to find out?
**Awareness/ Communication experiences**	7. How often do you receive routed documentation of preoperative assessments?	Is routing the documentation to the PCP a useful way of informing them about surgeries?	Is this documentation useful to you (or would it be useful)? How does/would it influence patient care?
**Utility of being able to communicate**	8. In your opinion, how important is it to communicate with anesthesia and surgery about your patients prior to surgery?	Do PCPs want or need to communicate with anesthesia and surgery?	Are there certain situations when it would be important to be in touch with anesthesia and/or surgery?
**Perioperative communication experiences**	9. Do you know how to reach out to your patient's surgeon? How to reach their anesthesia provider?<br>10. Do you reach out to them directly or do communicate via a staff member?	Do PCPs currently have the ability to initiate communication with anesthesia/surgery?	Examples of times when you needed to talk to someone on the anesthesia team or when it might have been useful to be able to talk to them? To a surgeon?
	11. Can you think of any Examples of times you have spoken to an anesthesia provider? To a surgeon?<br>12. Have you encountered situations in which you have information you want to give to the	**Perioperative interdisciplinary communication experiences**	What happens more often: you reaching out to the surgeon or the surgeon reaching out to you?<br>Do you bill for the time you spend communicating with other providers?

	anesthesia or surgery team regarding your patient (before surgery)? If so, were you able to contact them? Please elaborate.		
**Communication preferences**	13. What are the preferred channels of communication for you and your office with other healthcare providers? (eg. Fax, telephone, email, mail, EMR)<br>14. Which channels of communication do you prefer to avoid, if any?	**How do primary care providers prefer to communicate?**	How do you prefer to send info to other providers?<br>How do you prefer to request info?<br>Which channel of communication do you prefer to use to discuss a complex patient?
	15. DHMC Anesthesia providers hold weekly multidisciplinary meetings about complex older patients, which include a psychiatrist, geriatrician, surgeon and geriatric nurse/care coordinator. What do you think about primary care doctors participating in these preoperative multidisciplinary meetings about shared complex or high-risk patients?	Should we invite PCPs to participate in multidisciplinary meetings?	Have you ever received routed documentation of these meetings?<br>Would you be interested in being invited to participate in meetings about your patients?
	16. What are the main barriers that impede communication with anesthesia providers and surgeons?	Communication barriers	Can you think of anything that could be done to facilitate bidirectional communication?
	17. How could the perioperative clinic communicate with you during or after the pre-operative evaluation?<br>18. What kind of information would be most helpful/important to you? (eg. nature/ timing of surgical procedure, implications for future functional status, need for prehabilitation or preparation, medication changes, testing or rehabilitation, new diagnoses uncovered during preop assessment, positive screen for cognitive impairment, nutritional status concerns, changes in advanced directives or goals of care)	**How should we communicate with primary care?**	What kind of information do you have that you think would be most valuable to the anesthesia team? (eg. up to date medication list, degree of optimization of comorbid conditions, current cognitive and functional status, results of lab/imaging tests, details of complex social situations, history of anesthesia related complications, undocumented conversations regarding advanced care planning)

**Ideas and potential strategies to enhance communication**	19. What do you wish was in place to facilitate communication with anesthesia and surgery providers before surgery?	**To identify the most promising strategies to optimize communication**	Do you have any suggestions or things you think should be done?
	20. What do you think about the option of being able to generate a medical summary via the EHR (Most recent vitals, weight, height, problem list, medication list, most recent lab and imaging results) in order to convey information to the anesthesia and surgery teams?		What do you think about filling out a preoperative assessment form via fax or email?<br>What do you think about secure medical texting?
**Preoperative assessment and optimization**	21. Do you see your patients for a preoperative appointment?<br>22. What is your primary goal for the preoperative appointment?	PCP perspective of preoperative assessment	Examples of things you do with your patients during a preoperative appointment?
	23. What do you think is the anesthesiologists' responsibility in the medical and psychosocial evaluation and preparation before surgery?<br>24. What do you think is the responsibility of the surgeon and their office in the medical and psychosocial evaluation and preparation for surgery?	Opinions on the distribution of preoperative responsibilities between anesthesia, primary care and surgery. PCP perceptions of anesthesia preoperative assessment	How do you perceive your own role in the medical and psychosocial evaluation and preparation before surgery?
**Concluding/Final thoughts**	25. Are there any other thoughts you would like to share about perioperative communication between PCPs, anesthesia providers and surgeons?		

Surgeon Interview Guide

Thank you for your time and willingness to be interviewed to provide your valuable experience and insights for this research study. Is it ok for me to begin recording now?

Category	Interview Question	Relevant Objective	Follow-up Questions
**General questions about the interviewee**	1. How long have you been practicing?<br>**2.** Do you or have you practiced in other places in the past?	If yes, for subsequent questions explore how experiences have differed in other practice settings/locations	Which other places/settings?
**Perioperative communication experiences with PCPs**	3. How much time do you typically spend on gathering information about patients during preoperative assessment?<br>4. How often do you communicate with primary care providers?<br>a. How often do you reach out for information about a patient prior to surgery?<br>b. How do you convey information to PCPs within and outside of the DH system?<br>5. Do you contact them directly or do you ask a staff member to contact them?<br>6. How often are you able to "close the loop" when communicating with the perioperative clinic or PCPs and how is this most frequently achieved?<br>7. What challenges do you encounter when trying to convey or obtain information from primary care providers?<br>8. Have you ever been contacted by a PCP before surgery? If yes, please elaborate.	**Perioperative interdisciplinary communication experiences**	(2)Can you think of any examples of times you needed to communicate with PCPs?<br>Examples of times when it would have been helpful to communicate with a PCP but you were unable to connect?<br>(3) How often do you speak with them directly?<br>(6)What happens more often: you reaching out to the PCP or the PCP reaching out to you?

**Perioperative communication experiences with anesthesia**	9. How often do you communicate with anesthesia providers from the preoperative clinic?<br>10. Do you contact them directly or do you ask a staff member to contact them? How often do you speak with them directly?<br>11. How often do you speak with anesthesia providers in pre-operative area immediately before surgery?<br>12. How do you convey information to the anesthesia team?<br>13. What challenges do you encounter when trying to convey or obtain information from anesthesia providers?	**Perioperative interdisciplinary communication experiences**	Can you think of any examples of times you talked to or corresponded with anesthesia providers during preoperative assessment that were positive or negative experiences?<br>Examples of times when it would have been helpful to communicate with an anesthesiologist but you were unable to connect?<br>Do you bill for the time you spend communicating with other providers?
**Communication preferences**	14. What are the preferred channels of communication for you with other healthcare providers? (eg. Fax, telephone, email, mail, EMR)<br>15. Which channels of communication do you prefer to avoid, if any?	**How do surgeons prefer to communicate?**	How do you prefer to send info to other providers?<br>How do you prefer to request info?<br>Which channels of communication do you prefer to use to discuss a complex patient?
	16. Do you think it is important to communicate with the PCP before surgery?<br>17. Which pieces of information do you perceive as most important to convey to PCPs?<br>18. Which pieces of information do you perceive as most important to receive from PCPs?		What kind of information do you think would be most valuable to the PCP?
	19. DHMC Anesthesia providers hold weekly multidisciplinary meetings about complex older patients, which include a psychiatrist, geriatrician, surgeon and geriatric nurse/care coordinator. What do you think about primary care doctors participating in preoperative multidisciplinary meetings about complex or high-risk patients?	Should we invite PCPs to participate in multidisciplinary meetings?	

	20. What are the main barriers that impede communication with anesthesia providers and PCPs?<br>21. What do you wish was in place to facilitate communication with anesthesia and primary care providers before surgery?	Communication barriers	What might make it easier to communicate?<br>Do you have any suggestions or things you think should be done?
**Preoperative assessment and optimization**	22. How do you perceive the perioperative clinic's responsibilities in preoperative assessment, optimization and preparation?<br>23. How do you perceive the PCP's responsibilities in preoperative assessment, optimization and preparation?	Surgeon perspective of preoperative assessment, distribution of preoperative responsibilities between anesthesia, primary care and surgery.	Examples of things you do with your patients during a preoperative appointment?
**Concluding/Final thoughts**	Are there any other thoughts you would like to share about perioperative communication between PCPs, anesthesia providers and surgeons?		
