# Supplementary material for: Routine preoperative assessment for cataract surgery is a source of frustration for primary care providers
Source: BMC Health Serv Res. 2024 Sep 17;24:1087. doi: 10.1186/s12913-024-11484-0 (PMC11409469; doi:10.1186/s12913-024-11484-0)
Supplement: Supplementary file 2 — Supplementary Material 2 [file 12913_2024_11484_MOESM2_ESM.docx]

**COREQ (COnsolidated criteria for REporting Qualitative research) Checklist**

A checklist of items that should be included in reports of qualitative research

| **Topic** | **Item No.** | **Guide Questions/ Description** | **Author Responses** |
| --- | --- | --- | --- |
| **Domain 1: Research team and reflexivity** | | | |
| *Personal Characteristics* | | | |
| Interviewer/facilitator | 1 | Which author/s conducted the interview or focus group? | All interviews were conducted by the first author (DR), an anesthesiologist-researcher with training and experience in qualitative methods. (Please see Methods section) |
| Credentials | 2 | What were the researcher’s credentials? E.g. PhD, MD | MD |
| Occupation | 3 | What was their occupation at the time of the study? | T32 Clinical Research Fellow |
| Gender | 4 | Was the researcher male or female? | Female |
| Experience and training | 5 | What experience or training did the researcher have? | Training – courses at the Dartmouth Institute, ResearchTalk  Experience – anesthesia and preoperative assessment (8 years), qualitative research (2 years) |
| *Relationship with participants* | | | |
| Relationship established | 6 | Was a relationship established prior to study commencement? | A relationship was not established prior to study commencement. |
| Participant knowledge of the interviewer | 7 | What did the participants know about the researcher? e.g. personal goals, reasons for doing the research | Participants were briefed on the interviewer's background and the objectives of the research. (Please see the Methods section) |
| Interviewer characteristics | 8 | What characteristics were reported about the inter-viewer/facilitator? e.g. Bias, assumptions, reasons and interests in the research topic | Participants were informed of the interviewer’s background as an anesthesiologist interested in perioperative medicine and geriatric surgery, who was conducting a study with the objective of improving inter-specialty communication. |
| **Domain 2: Study design** | | | |
| *Theoretical framework* | | | |
| Methodological orientation and Theory | 9 | What methodological orientation was stated to underpin the study? e.g. grounded theory, discourse analysis, ethnography, phenomenology, content analysis | The theoretical framework for the qualitative analysis incorporated core principals from the phenomenology and case study qualitative traditions. (Please see the Methods section) |
| Sampling | 10 | How were participants selected? e.g. purposive, convenience, consecutive, snowball | Using consecutive sampling, clinicians who expressed willingness were contacted to schedule an in-person or virtual interview. (Please see the Methods section) |
| Method of approach | 11 | How were participants approached? e.g. face-to-face, telephone, mail, email | Eligible clinicians were invited by email or phone to participate in an online preoperative communication survey. The survey included a question regarding willingness to be interviewed. Clinicians who expressed willingness were contacted by email or phone to schedule an interview. (Please see the Methods section) |
| Sample size | 12 | How many participants were in the study? | Twenty clinicians were interviewed: four anesthesia, seven surgery, and nine PCPs.(Please see the Results section) |
| Non-participation | 13 | How many people refused to participate or dropped out? Reasons? | Participation was voluntary – those who were not interested in being interviewed did not respond “yes” the willingness question in the survey and were not contacted. |
| *Setting* | | | |
| Setting of data collection | 14 | Where was the data collected? e.g. home, clinic, workplace | Interviews took place in person, over the telephone, or via videoconferencing, depending on clinician preference. (Please see the Methods section) |
| Presence of non-participants | 15 | Was anyone else present besides the participants and researchers? | Nobody else was present. |
| Description of sample | 16 | What are the important characteristics of the sample? e.g. demographic data, date | The PCPs in our study sample ranged in age between 30 and 60+ and included both male and female participants. PCP specialties included internal medicine, family medicine, and geriatrics. Of the nine PCPs who participated in the parent study, six (one advanced practice provider and five physicians) brought up the frequent preoperative assessments they perform for cataract procedures and were included in the current manuscript. (Please see Results section) |
| *Data collection* | | | |
| Interview guide | 17 | Were questions, prompts, guides provided by the authors? Was it pilot tested? | Yes, please see the Methods section for more details. |
| Repeat interviews | 18 | Were repeat interviews carried out? If yes, how many? | No repeat interviews were conducted. |
| Audio/visual recording | 19 | Did the research use audio or visual recording to collect the data? | Yes, WebEx videoconferencing software was used to record and transcribe all interviews. (Please see the Methods section) |
| Field notes | 20 | Were field notes made during and/or after the interview or focus group? | Yes, following each interview, field notes were taken to document the interviewer's reflections on the general atmosphere, any technical issues or disruptions encountered, and the main takeaways. (Please see the Methods section) |
| Duration | 21 | What was the duration of the interviews or focus group? | The duration was between 32 and 86 minutes (mean interview time = 47 minutes). (Please see the Methods section) |
| Data saturation | 22 | Was data saturation discussed? | Data saturation was not discussed because we interviewed all willing clinicians and did not base our sample size on an attempt to reach saturation. (Please see the Methods section) |
| Transcripts returned | 23 | Were transcripts returned to participants for comment and/or corrections? | To respect their time, we did not request comments or corrections of the transcripts from clinicians. |
| **Domain 3: analysis and findings** | | | |
| *Data analysis* | | | |
| Number of data coders | 24 | How many data coders coded the data? | Coding was performed by 3 researchers. (Please see the Methods section) |
| Description of the coding tree | 25 | Did authors provide a description of the coding tree? | Codes were organized by category to help manage the data but a coding tree was not part of our analysis methodology. |
| Derivation of themes | 26 | Were themes identified in advance or derived from the data? | Themes were derived from the data (Please see the Methods section). |
| Software | 27 | What software, if applicable, was used to manage the data? | Microsoft office and Atlas.ti were used to manage the data (Please see the Methods section). |
| Participant checking | 28 | Did participants provide feedback on the findings? | To respect their time, we did not request feedback on the findings from clinicians. |
| *Reporting* | | | |
| Quotations presented | 29 | Were participant quotations presented to illustrate the themes/findings?  Was each quotation identified? e.g. participant number | Yes, please see the Results section and Table 1. |
| Data and findings consistent | 30 | Was there consistency between the data presented and the findings? | Yes, the findings were guided by the interview data (please see the Results section). |
| Clarity of major themes | 31 | Were major themes clearly presented in the findings? | Yes, please see the Results section. |
| Clarity of minor themes | 32 | Is there a description of diverse cases or discussion of minor themes? | The themes in this study could be considered subthemes derived from the parent study’s major theme regarding PCP frustration over frequent referrals for preoperative assessment for cataract surgery.(Please see Methods and Results sections) |

Developed from: Tong A, Sainsbury P, Craig J. Consolidated criteria for reporting qualitative research (COREQ): a 32-item checklist for interviews and focus groups. *International Journal for Quality in Health Care*. 2007. Volume 19, Number 6: pp. 349 – 357
